# Identification and Functional Analysis of *Cynoglossus semilaevis* Z-Linked E3 Ubiquitin Ligase *rnf34*

**DOI:** 10.3390/ani14020311

**Published:** 2024-01-19

**Authors:** Lu Li, Xihong Li, Yadong Chen, Yingming Yang, Na Wang, Wenteng Xu

**Affiliations:** 1 School of Fishery, Zhejiang Ocean University, Zhoushan 316022, China; 2 Function Laboratory for Marine Science and Food Production Process, Laoshan Laboratory, Yellow Sea Fisheries Research Institute, Chinese Academy of Fishery Sciences (CAFS), Qingdao 266071, China

**Keywords:** *Cynoglossus semilaevis*, ubiquitin ligase, *rnf34*, spermatogenesis, siRNA-mediated knockdown

## Abstract

**Simple Summary:**

There is sex dimorphism in *Cynoglossus semilaevis*. And the female *C. semilaevis* has reversed to the pseudomale, hindering the development of the industry. The growth state of pseudomale is similar to male *C. semilaevis.* However, the pseudomales only produce Z-sperm, with W-sperm specifically disappearing. As transcriptome technology has developed, we have begun to investigate the function of E3 ubiquitin ligase in the ubiquitination pathway during spermatogenesis. In this study, we have cloned and described the *C. semilaevis* Z-linked E3 ubiquitin ligase *rnf34*. Its effect might begin in the early development of gonads, which is mainly localized in germ cells. These data indicate its role in gametogenesis, which will provide new genetic resources for the mechanism of abnormal spermatogenesis in fish pseudomale as well as exploratory control techniques.

**Abstract:**

The high proportion of males in *C. semilaevis* hinders their industrial development. The genetic ZW individual can become a pseudomale by sex reversal. And the pseudomale can produce Z-sperm (with epigenetic information to cause sex reversal) while W-sperm is absent, which leads to an even higher male proportion in offspring. Recently, with the development of transcriptomic technologies, research on spermatogenesis in *C. semilaevis* has been focused on the ubiquitination pathway. In this study, we analyzed the function of the ubiquitin ligase *rnf34* gene on the Z chromosome. A qPCR experiment showed that its expression level in the gonad was the highest among different tissues. In the ovary, the expression gradually increased with development from 40 days post-hatching (dph) to 1.5 years post-hatching (yph). In the testis, *rnf34* showed increased expression from 40 dph to 6 months post-hatching (mpf) and stabilized up until 1.5 ypf. In situ hybridization showed that the mRNA of *rnf34* was mainly distributed in the germ cells of the testis and the ovary. In vivo siRNA-mediated knockdown of the *rnf34* gene in male fish affected the expression of a series of genes related to sex differentiation and spermatogenesis. These results provide genetic data on the molecular mechanisms of gonadal development and spermatogenesis in *C. semilaevis*.

## 1. Introduction

Males and females of many species have markedly different morphological sizes, and this sexual dimorphism may have a clear advantage over other species when it comes to survival. For example, in *Lophius litulon*, the female individuals are ten-times larger than the males. Under the same breeding conditions, male *Pelteobagrus fulvidraco* grows about 2.5-to-3 times as fast as the female [1]. In addition, as one of the most important economic species in China, *Cynoglossus semilaevis*, has a heterogametic sex determination (ZW/ZZ) system and exhibits great sex dimorphism. Mature females are two-to-four times bigger than mature males [2]. Thus, the economic benefits could be greatly improved if the proportion of females in the breeding population could be increased. However, genetic females (ZW) could be sex-reversed to phenotypic males (named pseudomales). Pseudomale fish have similar physiological functions to normal male fish that can produce sperm and offspring [3,4]. However, the sperm produced by pseudomales contains only Z type, and the epigenetic information results in its offspring more prone to be pseudomale [5]. Due to these reasons, the male proportion can even reach to 80–90% of the entire community, seriously hindering the development of the industry [6].

Some researchers have tried to obtain WW hypergamy by female nuclear development or breeding pseudomales and females and have finally found that pseudomales could only produce Z spermatozoa [7,8]. Spermatogenesis is the process of spermatogonial stem cells maturing spermatozoa, which includes three stages: spermatogonial stem cell proliferation and differentiation, spermatocyte meiosis, and spermatozoa spherical deformation [9]. In this process, the generation of new cells and the apoptosis of old cells are in dynamic balance, which requires the participation of cellular, hormonal, genetic and epigenetic regulators, as well as the mutual regulation of various signals [10]. However, it is a strange and interesting phenomenon that Z spermatozoa can be produced normally while W spermatozoa “disappear”. The reason for this is still unclear. Early gonadal transcriptome analysis of *C. semilaevis* has shown that the regulators causing the differentiation and developmental differences between male and female gonads are mainly focused on the ubiquitination module [11]. Many other studies have also shown that ubiquitination plays a key role in spermatogenesis. For example, knockdown of the mouse ubiquitin ligase gene *rnf133* results in abnormal sperm heads and fertility defects [12]. The ubiquitin ligase TRIM36 helps to maintain normal fertilization and early embryonic development in *Xenopus* [13]. Mutation of the deubiquitinating enzyme Usp14 reduces testicular free monoubiquitin levels and leads to male sterility in *Drosophila* [14]. The deubiquitinating enzyme Uch-L1 regulates ubiquitin levels in *Monopterus albus* to maintain sex transition [15].

Throughout the pathway of ubiquitination, three enzymes work together to link ubiquitin to the target protein: ubiquitin-activating enzyme (E1), ubiquitin-binding enzyme (E2), and ubiquitin-ligase (E3) [16]. As the final enzyme step in the cascade, ubiquitin ligase E3 specifically recognizes its substrate for ubiquitination. It is also a major regulator during spermatogenesis [17,18]. In mammals, ubiquitin ligases play a wide range of roles in gonadal development and spermatogenesis, whereas their roles in fish have rarely been reported. In *C. semilaevis*, there are over 200 ubiquitin ligase genes, and while these candidates located on the Z chromosome are characterized in priority as Z-linked genes they are suggested to be closely related to male differentiation and spermatogenesis (*dmrt1*, *tesk1*, *neurl3*). Our previous comparison of sperm transcriptomes showed that six E3 ubiquitin ligases (including *neurl3*, *rchy1*, *dtx1, dtx3l-like rnf34,* and *trim25-like*) on the Z chromosome were differentially expressed between pseudomale and male *C. semilaevis* [19]. Among them, *rnf34* has mainly been studied in the immune aspects. For instance, Lateolabrax japonicus E3 ubiquitin ligase *rnf34* was recruited by the fish pathogen nervous necrosis virus (NNV) and takes part greatly in innate immunity. However, few reports about the role of *rnf34* in spermatogenesis have been published [20]. In this study, we identified an *rnf34* gene located on the Z chromosome and analyzed its sequence characterization, expression profile, cellular localization, and in vivo RNA interference effect in *C. semilaevis*. These data suggest its role in sex differentiation and spermatogenesis, which will provide new resources for exploring the mechanism of pseudomale abnormal spematogenesis and developing sexual control techniques in fish.

## 2. Materials and Methods

### 2.1. Ethics Statement 

Under the approval number YSFRI-2021018, this study was conducted with the approval of the committee at the Yellow Sea Fisheries Research Institute. In the present study, fish individuals were anesthetized with MS-222 (20 mg/L dissolved in sea water) to reduce the pain of the fish prior to the experimental process. 

### 2.2. Preparation of Samples

*C. semilaevis* was received from Haiyang Aquaculture Experimental Base (Haiyang, China). Tail fins were clipped from each fish for genetic sex determination. The extracted DNA was used as a template for gender testing according to the DNA reagent extract instructions (TIANGEN Biotech, Beijing, China). PCR was performed with the primers Sex-F and Sex-R (Table 1), and the product was gel electrophoresis with 4% agarose concentration at 150 V voltage for 25 min. At the end of electrophoresis, there were two bands (134 bp and 169 bp) amplified in females and one band (169 bp) in males [21]. In this study, spleen, intestine, gills, kidney, liver, and gonad samples were collected from males and females 1.5 years of age, fast frozen in liquid nitrogen, and stored at −80 °C. Gonads from *C. semilaevis* at different developmental stages, including 40 days, 60 days, 90 days, 6 months, and 1.5 years post hatching (40 dph, 60 dph, 90 dph, 6 mph and 1.5 yph), were also picked up. Each stage contains three males and three females. The gonad samples were stored in −80 °C refrigerators for RNA extraction. At the same time, parts were preserved in 4% (*w*/*v*) paraformaldehyde fixing solution and paraffin sectioned for in situ hybridization (ISH). 

### 2.3. Gene Sequence Analysis and Alignment

Based on the sequence from NCBI (https://www.ncbi.nlm.nih.gov/gene/103398329) (accessed on 16 October 2023), primers *rnf34* F and *rnf34* R (Table 1) were designed for the cloning and validation of *C. semilaevis rnf34*. The encoded amino acid and the open reading frame (ORF) were analyzed using Clustal Omega (http://www.ebi.ac.uk/Tools/msa/clustalo/) (accessed on 18 October 2023). Using Exspasy (https://web.expasy.org/compute_pi/) (accessed on 20 October 2023), we calculated the molecular weight (Mw) and theoretical pI. The conserved domain was predicted with SMART (http://smart.embl-heidelberg.de/) (accessed on 20 October 2023). A phylogenetic tree was conducted via MEGA 11.0 software with the neighbor-joining method. The species used to construct the evolutionary tree in this experiment are the following species: Paralichthys olivaceus XM_020105429.1; Pleuronectes platessa XM_053420090.1; Scophthalmus maximus XM_035640571.2; Acanthochromis polyacanthus XM_022220822.2; Stegastes partitus XM_008302041.1; Labrus bergylta XM_020633041.2; Sparus aurata XM_030417831.1; Anarrhichthys ocellatus XM_031849398.1; Cyclopterus lumpus XM_034541976.1; Monopterus albus XM_020590646.1; Sphaeramia orbicularis XM_030143366.1; Cynoglossus semilaevis XM_025053151.1; Xenopus laevis XM_018244826.2; Chelonia mydas XM_037878336.2; Gallus gallus NM_001396334.1; Mus musculus NM_001417908.1; Homo sapiens NM_194271.3; Sus scrofa XM_021073434.1.

### 2.4. Gene Expression Pattern Analysis

Trizol (Invitrogen, Carlsbad, CA, USA) was used for RNA extraction from different tissues. The experimental materials used throughout the process had to be sterilized. A PrimeScript™ RT reagent Kit with gDNA Eraser (TaKaRa, Otsu, Japan) was employed for cDNA preparation. The reverse transcription kit was used in two steps according to the instructions. The first step was to remove the genomic DNA. The second step was to reverse transcription, and the final product was stored at −20℃. Primers *rnf34* qF and *rnf34* qR (Table 1) were designed to analyze the expression patterns of this gene in different tissues and at various developmental stages. THUNDERBIRD^TM^Next SYBR^®^qPCR Mix (TOYOBO, Osaka, Japan) and the real-time fluorescent quantitative PCR system (AppliedBiosystems, Foster City, CA, USA) were used for qPCR. The following protocol was set for qPCR: 95 °C for 30 s, 95 °C for 5 s, 60 °C for 30 s, then 40 cycles are performed. *β-actin* was used as the internal reference gene, and each group was performed in three replicates. The experimental data were analyzed by the 2^−ΔΔct^ method to calculate the expression levels of the gene. SPSS 26.0 was used for multiple comparative analyses of the data, and a *p*-value less than 0.05 was considered to indicate a significant difference between the two groups. In multiple comparisons, a, b, and c are groups of different levels of differential expression.

### 2.5. Cloning, Characterization, and Activity Detection of the rnf34 Promoter

The promoter region of *rnf34* was first amplified with primers *rnf34* pF and *rnf34* pR (Table 1). pGL3-basic vector (Promega, Madison, WI, USA) performed single enzyme digestion with HindIII restricted endoenzyme digestion. The promoter fragment was inserted into a incised pGL3-basic vector (Promega, Madison, WI, USA) to obtain pGL3-rnf34 by using the TOROIVD^®^ One Step Fusion Cloning Mix seamless cloning kit (TOROIVD, Jangsu, China). After having the sequencing results were successfully compared with the sequences in NCBI, the plasmid was extracted using the small and medium extraction kits without endotoxin (TIANGEN Biotech, Beijing, China). pGL3-control was used as a positive control, while pGL3-basic was used as a negative control. Human embryonic kidney (HEK) 293 T cells were preserved in DMEM/F-12 medium containing 10% fetal bovine serum (FBS, Gibco, New York, NY, USA) and 1% bFGF (Invitrogen, Carlsbad, CA, USA) under 5% CO_2_ and 37 °C. HEK 293 T cells were transfected with pGL3-rnf34, pGL3-control, and pGL3-basic by using Lipo8000TM transfection reagent in 24-well plates, respectively, with each well receiving 800 ng of plasmid. As the internal reference, 40 ng of the pRL-TK plasmid was used per well. By using Dual Luciferase Reporter Gene Assay Kits (Beyotime, Shanghai, China), the luciferase activities of these cells were determined after 48 h. The SPSS 26.0 program was used to conduct a least significant difference (LSD) statistical analysis. With the online tools PROMO (https://alggen.lsi.upc.es/cgi-bin/promo_v3/promo/promoinit.cgi?dirDB=TF_8.3/) (accessed on 20 October 2023) and JASPAR (http://jaspar.genereg.net/) (accessed on 20 October 2023), possible transcription factors for the rnf34 promoter were predicted and screened. 

### 2.6. Cellular Localization of rnf34 mRNA in Gonads

Primer pairs *rnf34* probe F and probe R with T7 polymerase and SP6 endonuclease sites were designed (Table 1) to amplify a 448 bp fragment. The PCR products were recovered. In vitro transcription by T7 or SP6 RNA polymerases was performed to generate digoxin (DIG)-labeled antisense or sense RNA probes according to the kit (Roche in vitro transcription) and the digoxin probe instructions. After that, the probe was purified. The gonad samples of 1.5 yph male and female fish were sliced and fixed after dewaxing and hydration. Sections were pre-hybridized for 4 h at 60 °C. In identical liquid, an overnight incubation at 60 °C with probes (final concentration 0.2 g/mL) was performed on gonadal slices. Incubation with anti-DIG antibodies (Roche) was performed overnight after slices were blocked for 4 h at room temperature. In the end, nitroblue tetrazolium/5-bromo-4-chloro-3-indolyl phosphate (Roche, Mannheim, Germany) was used to generate the signal. Photos were taken with a Nikon EClIPSE 80i microscope equipped with 40×, 100×, and 200× lenses.

### 2.7. In Vivo RNA Interference (RNAi) for rnf34 and the Effect on Other Genes

Three siRNAs targeting different sites of *rnf34* and the negative control (NC) were synthesized by Sangon (Shanghai, China). Non-targeted siRNA was used as a negative control. The *C. semilaevis* gonad cell lines were established in the laboratory by isolating the gonads. The established testicular and ovarian cells lines (derived from testes and ovaries and composed mainly of somatic cells) used for RNAi were performed. The cells were cultured in an incubator at 24 °C and lived in the L-15 medium containing 20% fetal bovine serum (FBS), bFGF (5 ng/mL), 5 ng/mL EGF, 27.5 μmol/L beta-mercaptoethanol, and 5% triple antibody (Beyotime, Shanghai, China). It was then time to carry out the subculture once the cells filled the culture bottle. When the isodensity reached 80–90% again, the cells were spread into the 12-well cell culture plate. The transfection of gonadal cell lines with siRNA interference was conducted with a CP Regent transfection kit (RiboBio, Guangzhou, China). The siRNA group and the NC group experiments were repeated three times. RNA isolation and qPCR analysis were carried out as mentioned above to measure the expression level of *rnf34* in the cells. In the RNAi experiment in cell lines, 48–72 h was recommended according to the instructions of the CP Regent transfection kit. Our pretrial showed that 72 h has the best knockdown effect. The one with the lowest expression level was considered to have the best knockdown effect and was selected for injection into 90 dph *C. semilaevis*. As part of the in vivo RNAi experiments, the preferred siRNA concentration of 0.05–0.25 nmol/g, determined based on Raybiotech recommendations with a few modifications, was injected into the gonadal location (0.025 nmol/g injection concentration) by microsyringe. These *C. semilaevis* were equally divided into negative control (NC) and experimental groups (4 individuals in each group). At 72 h after the injection, the gonads were taken for gene expression analyses of *rnf34* and other sex-related genes including *sox9*, *sox9-a*, *cyp19a*, and *neurl3*, with the above described methods. The primers that were used for the experiment are shown in Table 1.

## 3. Result

### 3.1. Sequence Verification and Evolutionary Tree Analysis of rnf34 in C. semilaevis

**Figure 1:**
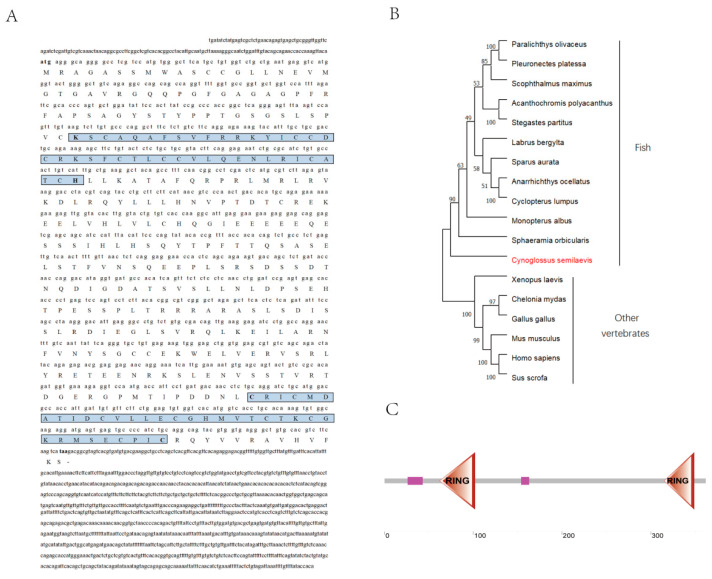
Sequence verification and phylogenetic tree analysis of *C. semilaevis rnf34*. (**A**) mRNA sequence and amino acid sequence of *rnf34.* The blue region is a ring domain. (**B**) A total of 17 species *rnf34* sequences constitute a phylogenetic tree. *C. semilaevis* is highlighted in red to show its evolutionary position. (**C**) Predicted protein structures of *rnf34*. Horizontal gray bars represent amino acid sequences with no predictive functional domains, while colored boxes represent regions with reliable predictive functional domains. Two zinc finger RING type domain (red triangle); two low complex regions (pink rectangle).

There are 7 exons and 6 introns in the *C. semilaevis rnf34* gene (GenBank number 103398329), which is located on the Z chromosome. The coding region of *rnf34* mRNA consisted of 1089 bases, the 5’ UTR of 156 bases, and the 3’ UTR of 1264 bases. The protein was deduced with 362 amino acids, while its predicted molecular mass is 40.37 kDa and its isoelectric point is 7.04 (Figure 1A). Based on conserved domain prediction (Figure 1C), this protein consisted of two zinc finger RING type domains (aa 62–102 and aa 315–349).

Phylogenetic analysis revealed that vertebrate *rnf34* sequences were divided into two groups. One group was formed by *C. semilaevis rnf34* and other fish *rnf34*, while the other group was formed by other vertebrate *rnf34* (Figure 1B).

### 3.2. Expression Patterns of rnf34 in Different Tissues and Sexes

**Figure 2:**
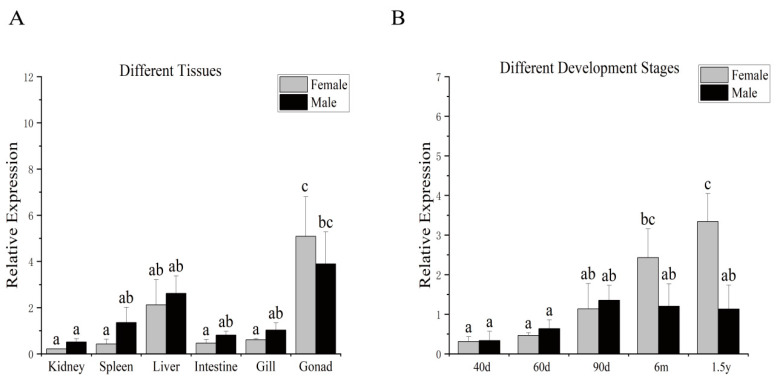
The expression pattern of *C. semilaevis rnf34* in gonads. (**A**) The expression pattern of *rnf34* in different tissues. (**B**) The expression pattern of *rnf34* in different periods of gonadal development. In multiple comparisons, different alphabets indicated significant difference.

qPCR analysis revealed that *rnf34* mRNA was widely distributed in all detected tissues of both females and males, with the highest expression levels found in the gonads (Figure 2A). *rnf34* expression was detected at all developmental stages. In the testis, the expression of *rnf34* gradually increased from 40 dph to 90 dph and stabilized after reaching the highest level at 90 dph (Figure 2B). In the ovary, the expression of *rnf34* increased with developmental stage and reached a peak at 1.5 yph. 

### 3.3. Promoter Structural Analysis and Activity Detection of C. semilaevis rnf34

**Figure 3:**
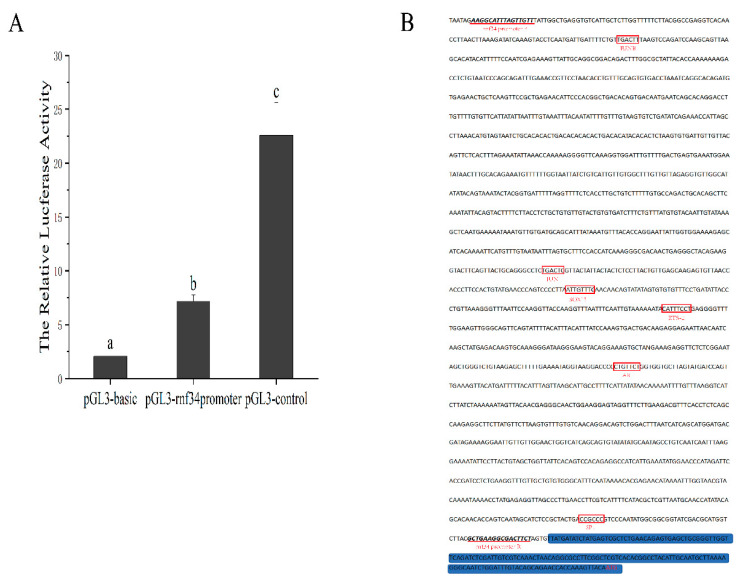
Promoter activity and structure analysis of *C. semilaevis rnf34.* (**A**) pGl-rnf34 promoter transfected into HEK 293 T cells showed significant activity compared with pGl-basic (negative control) and pGl-control (positive control). In multiple comparisons, different alphabets indicated significant difference. (**B**) Prediction of transcription factor binding sites marked by a red box on the promoter. The blue region is the 5’UTR of *rnf34*.

A 1992 bp promoter was cloned in the upstream region of *rnf34* 5’UTR. After cell transfection and dual luciferase activity detection in HEK 293T cells, the pGL3-rnf34 recombinant plasmid-transfected cells were observed to have significantly higher firefly/Renilla luciferase activity than the pGL3-basic transfected group. The results showed that the amplified promoter sequence of *rnf34* had obvious activity in initiating gene expression in the cell (Figure 3A). Furthermore, by utilizing the PROMO and JASPAR analysis, the numerous binding sites for transcription factors associated with apoptosis were anticipated within the *rnf34* promoter sequence, encompassing specificity protein (SP1), Ets2 repressor factor (Ets-2), jun proto-oncogene (JUN), and jun B proto-oncogene (JUNB). In addition, the binding sites of male-related androgen receptor (AR) and SRY-box transcription factor 13 (SOX13) were also screened (Figure 3B).

### 3.4. Localization of rnf34 mRNA in Gonads

**Figure 4:**
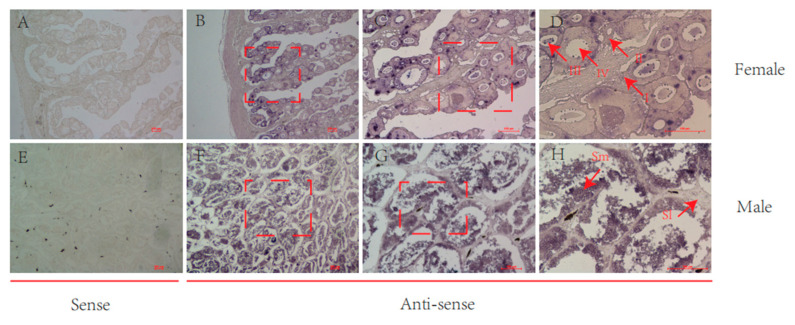
Cellular localization of *C. semilaevis rnf34* in 1.5 yph gonads. (**A**,**E**) Sense probes combined display no color. (**B**–**D**) Antisense probe binds to the ovary. Oocytes at different developmental stages are marked by I, II, III, and IV. (**F**–**H**) Antisense probe binds to the testis. Sm: sperm; Sl: seminal lobule. The red box indicates the area of the same section under different magnification.

According to the ISH, rnf34 was expressed primarily in the spermatozoa of the testis and in the oocytes of the ovary (Figure 4). It was expressed in oocytes at different stages of the ovary, especially in the late stage of the ovary. However, it was mainly expressed in sperm in the testis. Slices of fish from each sample are 1.5 years old. 

### 3.5. In Vivo RNAi-Mediated rnf34 Knockdown and Its Influence on the Expressions of Sex-Related Genes

**Figure 5:**
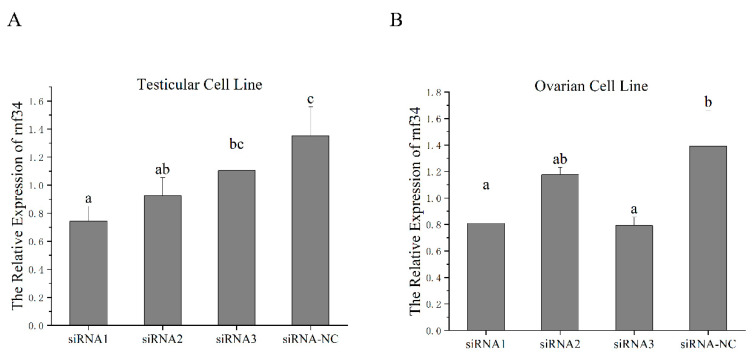
Design siRNAs to knockdown the gonadal tissue cells of *C. semilaevis.* (**A**) The relative expression levels of *rnf34* in the testicular cell line. (**B**) The relative expression levels of *rnf34* in the ovarian cell line. In multiple comparisons, different alphabets indicated significant difference. Groups with two letters represent no significant difference from groups with one of the letters.

**Figure 6:**
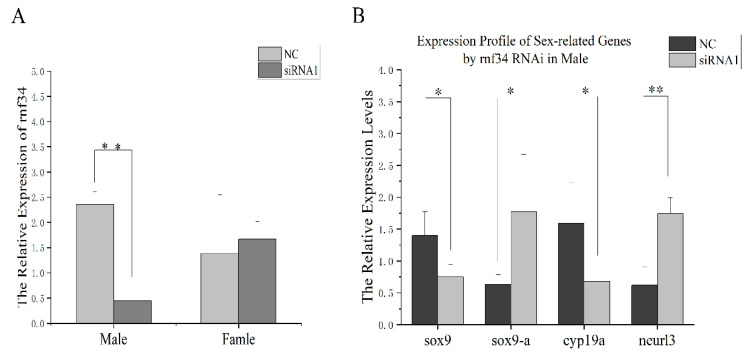
In vivo siRNA knockdown and sex-related gene expression levels. NC: the siRNA for *Nematoda*. (**A**) In vivo siRNA knockdown in males and females. (**B**) The effect of the knockdown of *rnf34* on other sex-related genes. One asterisk indicates a significant difference of less than 0.05, and two asterisks indicate a significant difference of less than 0.01.

According to the effect of the siRNAs in *C. semilaevis* cells, siRNA1 was selected for injection into the gonad of 90 dfh female and male fish (Figure 5). Compared with the NC group, the *rnf34* expression level was significantly decreased only in male fish (Figure 6A). The mRNA levels of *neurl3*, *sox9*, *sox9-a*, and *cyp19a* were also examined. A significant reduction was observed in the levels of *sox9* and *cyp19a* expression after RNAi. However, the expression levels of *sox9-a* and *neurl3* increased significantly (Figure 6B).

## 4. Discussion

Sexual growth dimorphism and sexual reversal make *C. semilaevis* a preferred species for investigating sex determination, gonadal differentiation, and sex control technology [22]. Interestingly, pseudomale tongue sole exhibit abnormal spermatogenesis, producing only Z sperm and no W sperm. The production of active Z sperm in pseudomales indicates that spermatogenesis can proceed until producing mature sperm, while W sperm might be absent during the process [9]. Triploid cyprinid fish can also develop round sperm cells, but these sperm cells are subsequently degraded. Transcriptome analysis of this triploid fish have shown that genes in the ubiquitin-related pathway are up-regulated, which triggered apoptosis and ultimately led to their sterility [23]. However, unlike triploid cyprinid fish, lacking a specific type of sperm is a unique phenomenon in vertebrate. What is the mechanism for W sperm absence and maintenance of Z sperm? In *C. semilaevis*, the gonadal and sperm transcriptomic analyses strongly suggest that the Z chromosome-linked genes and ubiquitination have an impact on spermatogenesis [11,19]. Our previous study has been focused on Z chromosome-linked E3 ubiquitin ligases, such as *neurl3*, *rchy1,* and so on [24,25]. In this study, we have characterized another Z-linked E3 gene, namely *rnf34*. The gene was widely distributed in different tissues with higher levels in the gonads. During different developmental periods of gonads, the expression of *rnf34* gradually increased in early development (from 40 dph to 90 dph), suggesting its role in the early development of *C. semilaevis* gonads. The expression in testis was rather stable after 90 dph. ISH results showed that *rnf34* was predominantly localized in germ cells, especially spermatocytes and spermatozoa. 

After siRNA-mediated knockdown in the testis, the expressions of several genes were influenced, such as up-regulation of *sox9-a* and *neurl3*, and downregulation of *sox9* and *cyp19a*. The expression of *sox9* and *sox9-a* was reported to be significantly up-regulated in adult males compared to adult females, which may be related to the formation of spermatogonia [26,27]. *cyp19a*, a cytochrome P450 aromatase, catalyzes the synthesis of estrogen from androgens and participates in ovarian differentiation [28]. It has been reported that *sox9-a* inhibits the expression of *cyp19a* by activating the Amh cascade effect [29]. Additionally, *neurl3* has been shown to be involved in spermatogenesis, and its expression is up-regulated after *rnf34* knockdown. These data together suggest that *rnf34* might play a negative role in spermatogenesis, or there may be a complementary regulation of the overall ubiquitination network. Spermatogenesis is a complex and dynamically regulated process that requires multiple parties to maintain it. The W chromosome has about one-third of the functional genes of the Z chromosome in terms of the genes coding for non-PAR predicted functional protein [7]. Is that why W sperms are less sustainable than Z sperms? If the phenotypic effects can be observed by long-term knockdown, the network of spermatogenesis and the disappearance of W sperms may be better explained. It is interesting that *rnf34* expression in the ovary increased from 40 dph to 1.5 yph and that it was mainly located in oocytes, while the role in female requires further investigation.

Rnf34 has been reported to be a ubiquitin ligase associated with caspase 8/10, which is synthesized as a key regulator to activate apoptosis [30,31]. In accordance, apoptosis signals were stronger in pseudomales than in males. Moreover, the *rnf34* promoter clone has an obvious activity, and it is expected that there are multiple transcription factor binding sites related to apoptosis, such as JUN and SP1 [32,33]. Whether W spermatocyte/spermatozoa undergo apoptosis needs further study. 

## 5. Conclusions

In summary, we have cloned and validated the *Cynoglossus semilaevis* Z-linked E3 ubiquitin ligase *rnf34*. The expression of *rnf34* gradually increased during early development (40–90 dph), indicating that its role began in early gonad development. ISH results showed that *rnf34* mRNA was mainly localized in germ cells. After siRNA-mediated knockdown, the expressions of *sox9-a* and *neurl3* were up-regulated, and the expressions of *sox9* and *cyp19a* were downregulated. These data suggest that *rnf34* may play a negative regulatory role in spermatogenesis. The regulatory effect in female fish remains to be studied. The activity of promoter verification indicates that the *rnf34* promoter is about 2000 bp upstream, and it predicted several transcription factor sites related to apoptosis which may have a regulatory effect on *rnf34*. In the cell apoptosis experiment, the fine apoptosis signal in the gonads of pseudomale fish was stronger; nevertheless, whether this was related to w sperm loss remains to be verified.

## Figures and Tables

**Table 1:** The primers or siRNAs used in present study.

Primer	Sequences (5′–3′)	Purpose	Product Size
*rnf34* F *rnf34* R	GATTGTCGTCAAACTAA ATGTATGTTCAGGTGTT	PCR	1430 bp
Sex-F Sex-R	CCTAAATGATGGATGTAGATTCTGTC GATCCAGAGAAAATAAACCCAGG	Sex identification	
*rnf34* qF *rnf34* qR	CCACTTATCCGCCCACC GCCGTTGAAAGGCTGTA	qPCR	189 bp
*sox9* qF *sox9* qR	AAGAACCACACAGATCAAGACAGA TAGTCATACTGTGCTCTGGTGATG	qPCR	150 bp
*sox9-a* qF *sox9-a* qR	GACCAAGTGTGTAATGTGACCAAG GCTCTTGGTGTTGTTATATCCACG	qPCR	227 bp
*cyp19a* qF *cyp19a* qR	GGTGAGGATGTGACCCAGTGT ACGGGCTGAAATCGCAAG	qPCR	230 bp
*neurl3* qF *neurl3* qR	CTGGTGTTTAGCAGCCGTCCT CCAGAACTCCAGCACTGACCC	qPCR	234 bp
*β-actin* F *β-actin* R	CCTTGGTATGGAGTCCTGTGGC TCCTTCTGCATCCTGTCGGC	qPCR	150 bp
*rnf34* probeF *rnf34* probeR	ATTTAGGTGACACTATAGAATGCGTCTTAGAGTAAAGGA TAATACGACTCACTATAGGGGCACCCTGAATAATTGACAAAGTTC	ISH	448 bp
*rnf34* pF *rnf34* pR	AAGGCATTTAGTTGTT AGAAGTCGCCTTCAGC	promoter cloning	1992 bp
siRNA1	GCACCCAGUGCUGGAUAUUTT AAUAUCCAGCACUGGGUGCTT	siRNA site1	
siRNA2	CCAUCUGCAAUGGAACUAUTT AUAGUUCCAUUGCAGAUGGTT	siRNA site2	
siRNA3	GGAUGUGACGCUGAGUGAUTT AUCACUCAGCGUCACAUCCTT	siRNA site3	
siRNA-nc	UUCUCCGAACGUGUCACGUTT ACGUGACACGUUCGGAGAATT	negative control	

## Data Availability

The original contributions presented in the study are included in the article, further inquiries can be directed to the corresponding author/s.
